# External validity of randomized clinical trial studying preventing depressive symptoms following acute coronary syndrome

**DOI:** 10.1002/brb3.2132

**Published:** 2021-06-17

**Authors:** Michael Tvilling Madsen, Knud Juel, Erik Simonsen, Ismail Gögenur, Ann Dorthe Olsen Zwisler

**Affiliations:** ^1^ Center for Surgical Science Department of Surgery Zealand University Hospital Koege Denmark; ^2^ National Institute of Public Health University of Southern Denmark Copenhagen Denmark; ^3^ Psychiatric Research Unit Slagelse Denmark; ^4^ Department of Clinical Medicine Faculty of Health and Medical Sciences University of Copenhagen Copenhagen Denmark; ^5^ REHPA ‐ Danish Knowledge Center for Rehabilitation and Palliative Care Odense University Hospital University of Southern Denmark Odense Denmark; ^6^ The Danish Clinical Quality Program (RKKP) Danish Cardiac Rehabilitation Database National Clinical Registries Aarhus Denmark

**Keywords:** acute coronary syndrome, depressive symptoms and anxiety, eligibility, external validity, randomized clinical trial

## Abstract

**Objective:**

The objective of the current study was to quantitatively explore aspects of external validity, both researcher's choices (eligibility) and patient's choices (consent), of a recently conducted clinical trial.

**Methods:**

A cohesive comparison between the MEDACIS trial (NCT02451293) database and a national quality and research database was conducted. Comparisons between both participants and nonconsenting patients (patient consent) and participants and noneligible patients (researcher selection) were performed. Comparisons of outcomes were depressive and anxiety symptoms, demographics, and somatic or psychiatric comorbidity.

**Results:**

Noneligible patients had significantly higher levels of depressive symptoms and anxiety and were older and more likely to suffer from unstable angina pectoris. Furthermore, noneligible patients were less likely to be married, had a lower educational level, used more medication, and had a higher frequency of comorbidity. Nonconsenting patients had significantly higher levels of depressive symptoms and anxiety and were older and more likely to be females compared to participants.

**Conclusion:**

Significant differences were present between noneligible patients and participants; however, more troublingly significant differences were shown between nonconsenting patients and participants. The presence of depressive symptoms and anxiety has a significant impact on patients’ willingness to give informed consent in clinical trials in cardiology with a focus on psychological outcomes.

## Graphical abstract

The current study highlights problems with the external validity of a randomized clinical trial in patients following acute coronary syndrome investigating psychological outcomes.This study shows that noneligible patients had significantly higher levels of depressive symptoms and anxiety.Nonconsenting patients had higher levels of depressive symptoms and anxiety and were older were more likely to be females compared to participants.The currents study's conclusion is limited by an incomplete matchup between the trial screening list and the cardiac rehabilitation clinical database.

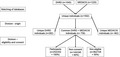

## 1 Significant Outcomes and Limitation

1. The current study highlights problems with the external validity of a randomized clinical trial in patients following acute coronary syndrome investigating psychological outcomes.
2. This study shows that noneligible patients had significantly higher levels of depressive symptoms and anxiety.
3. Nonconsenting patients had higher levels of depressive symptoms and anxiety and were older more likely to be females compared to participants.
4. The current study's conclusion is limited by an incomplete matchup between the trial screening list and the cardiac rehabilitation clinical database.

## 2 INTRODUCTION

Following acute coronary syndrome (ACS), approximately one in five patients will develop a depression that needs pharmacological treatment (Osler et al., 2016; Thombs et al., 2006). The significance of this clinical challenge has led to the international recommendation of screening for depression as part of cardiac rehabilitation (CR) (Lichtman et al., 2008).

Based on promising findings in the field of breast cancer (Hansen et al., 2014), the MEDACIS trial was initiated in 2016 in an attempt to prevent the development of depressive symptoms following ACS (Madsen et al., 2017). The randomized controlled trial (RCT) allocated patients to either 25 mg melatonin or a placebo once daily in a primary prophylaxis setup and followed the patient for 12 weeks. Eligible patients were contacted during their inpatient stay following ACS and if interested in participation included during first visit in the outpatient clinic during CR. Screening during recruitment was performed systematically and continuously to reflect flow of ACS patients at including centers. The MEDACIS trial ended up showing no effect of melatonin to prevent depressive symptoms; however, the no‐effect results could potentially be explained by limited external validity (Madsen et al., 2019). The current study was conducted to explore the external validity of the MEDACIS trial.

As in every clinical trial, a record of eligible and noneligible patients was kept. Likewise, eligible patients opting not to participate (nonconsenting) were recorded (Madsen et al., 2017). To distinguish further levels of the selection process, a distinction between biases related to the researcher's choices (noneligible patient) and patient's choice (nonconsenting) was made. In making this discrimination, we hoped to be able to describe different dimensions of the external validity of a randomized clinical trial in the field of psychiatric and cardiology research. From a clinical point of view, being able to make inferences about a given target population on the basis of a trial population is essential. In light of this, the external validity of a trial is paramount and should be reported as thoroughly as possible (Rothwell, 2005).

Based on a priori known observational and trial data, we expect noneligible patients to differ from trial participants by being older and possibly more likely to be females (Grace et al., 2004; Hansen et al., 2011; Hutchinson‐Jaffe et al., 2010; Sorensen et al., 2005). A priori no data were known with regard to nonconsenting patients; however, ideally, they should be similar to trial participants.

### 2.1 Aims of the study

We hypothesized that there would be a systematic difference in demographics and depressive symptoms and anxiety between trial participants and noneligible patients. On the contrary, we hypothesized that trial participants and nonconsenting patients were similar with regard to demographics and psychological outcomes.

## 3 MATERIAL AND METHODS

### 3.1 Study design

The basis of the analysis was data prospectively collected in the database from the MEDACIS trial (Madsen et al., 2017) and the Danish Cardiac Rehabilitation Database (DHRD) (Zwisler et al., 2016). The MEDACIS trial conducted from January 2016 until August 2017 in which 1,220 patients were screened, 492 were eligible, and 252 ended up being randomized (Madsen et al., 2019). In Denmark, patients following ACS participating in CR have been entered into the DHRD prospectively since August 2015, which overlaps with the conduct of the MEDACIS trial.

### 3.2 Setting

The MEDACIS trial was conducted at a total of five departments of cardiology with six associated cardiac outpatient clinics in Zealand, Denmark (supplementary material S1). The catchment area of including centers was an estimated 1.2 million citizens. The specific period of recruitment for each center is given in the supplementary material (S1). Eligible patients were contacted by a clinical trialist and introduced to the trial during their inpatient stay in relation to the ACS. A later inclusion meeting was planned at the outpatient clinic within 4 weeks as per the inclusion criteria. Data collection, exposure to melatonin, and follow‐up are all as presented in the published protocol for the MEDACIS trial (Madsen et al., 2017).

### 3.3 Participants

**FIGURE 1:**
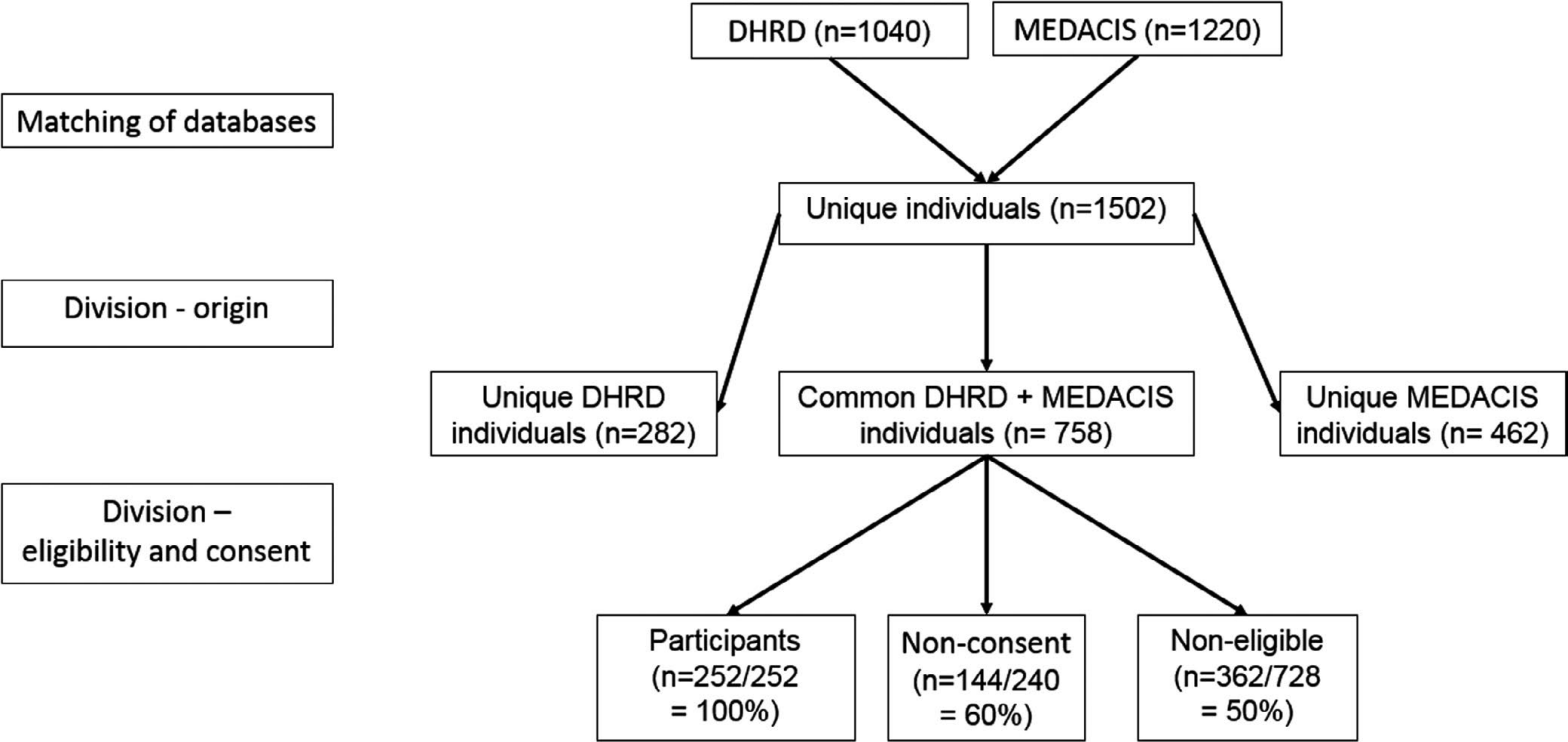
Cohort definitions and grouping. Matching based on center, recruitment period, and ACS diagnosis from MEDACIS screening list. Origin based on groupings of only in DHRD, overlap in DHRD + MEDACIS or only MEDACIS. Eligibility and consent based on MEDACIS CONSORT diagram, that is, 252 included in the MEDACIS trial, 240 patients who were eligible but did not give informed nonconsent, and 728 who were noneligible due to an exclusion criteria and hence excluded from the MEDACIS trial

Patients had to be admitted with an acute coronary syndrome at an including center during the period presented in the supplementary material (S1) to be eligible for the current study. An individual could be identified for this study from one of two sources, either the MEDACIS screening list or the DHRD. The screened patients from the MEDACIS trial (1,220) were matched against the corresponding source population from the DHRD (1,040), generated based on the center, recruitment period, and ACS diagnosis (Figure 1). Together, this yielded a sample size of 1502 unique patients. Based on the origin of data, 282 patients were unique to the DHRD database, 758 patients had an overlap with the MEDACIS screening list and the DHRD, and 462 were unique to the MEDACIS screening list (Figure 1). Based on eligibility and consent, the DHRD + MEDACIS cohort can be further divided into three groups: 252 participants, 144 in nonconsent group (144/240 = 60% completeness), and 362 in the noneligible group (362/728 = 50% completeness) (Figure 1).

The participants were followed for 12 weeks within both the MEDACIS trial and the DHRD, whereas the nonconsent and noneligible groups were included only in the DHRD. It should be noted that only patients participating in CR were included within the DHRD.

### 3.4 Variables

The primary demographic variables of interest for the comparisons between the databases of origin (Figure 1) were age, gender, and ACS diagnosis. In the analysis of comparisons based on the eligibility criteria and consent (Figure 1), the primary outcomes of interest were age, gender, ACS diagnosis, comorbidity, current antidepressant treatment, and hospital anxiety and depression score (HADS).

The HADS is divided into two subscores HADS‐anxiety (HADS‐A) and HADS‐depression (HADS‐D), both with a maximal score of 21 points from seven questions with a possible score of 0–3 (Zigmond & Snaith, 1983). As recommended, a cutoff of HADS‐A/D ≥8 was considered the threshold for anxiety or depression (Bjelland et al., 2002). Depression and anxiety were primary outcomes in the MEDACIS trial; therefore, intergroup comparisons on these psychiatric outcomes can be seen as primary outcomes of interest in the current study.

Exploratory demographic variables also for the comparison based on eligibility criteria and consent were civil status, educational level, smoking status, blood pressure, comorbidity, and medication. A full list of comorbidity variables and medication can be seen in supplementary material (S2).

### 3.5 Data sources

Age and gender were gathered at the point of inclusion into the DHRD or time of screening, depending on the source of the database. Likewise, ACS diagnosis was entered into the DHRD by the nurse at the initial meeting in the CR clinic or in relation to the screening by study investigators. Information on comorbidity and medication in the DHRD is gathered using data capture in national registers (e.g., national patient register (Lynge et al., 2011) and prescription database (Johannesdottir et al., 2012)) as described by Zwisler et al. (Zwisler et al., 2016). Similar information on comorbidity and medication was collected for the MEDACIS trial, as described in the protocol article (Madsen et al., 2017). Information on the civil status, educational level, smoking status, and blood pressure was gathered by nurse practitioners during CR and entered manually into the DHRD.

Screening by the HADS was conducted during the CR as per the guideline of the individual outpatient clinic. Patients either filled out the form during a visit to the CR or were given a copy to return on the following visit. At the time of the conduct of the MEDACIS trial, the HADS was not a key indicator of the DHRD; hence, entry into the database was not mandatory. If the HADS was missing, then the patient journal was searched for the entry of HADS data. If the patients included in the MEDACIS study had a missing value, the baseline HADS screening in the MEDACIS study was applied. This effort was made to reduce the amount of missing data. Either way, the HADS was collected; it was comparable with regard to content; however, the timing of the HADS could differ regarding the practice of the outpatient CR clinic (i.e., beginning, middle, or end of CR).

### 3.6 Statistical methods

Parametric or nonparametric statistics were applied depending on the distribution of data, which was assessed visually for each variable using qq‐plot. For demographic data, outcomes were reported as mean and standard deviation for continuous outcome and frequency and group percentages for categorical data. Comparisons between groups were all independent groups and performed according to the structure previously describe (Figure 1). In the case of a continuous variable, groups were compared using a *t* test, and the results were displayed as mean difference, 95% confidence interval, and a *p*‐value for the comparison. In the case of binary or categorical data, groups were compared using Fisher's exact test, and results were displayed as group frequency/percentage and a *p*‐value for the comparison.

At the initial level, we performed intergroup comparison on age, gender, and ACS diagnosis between groups of origin (DHRD, MEDACIS + DHRD, and MEDACIS only—Table 1). In the second step of analysis, we focused solely on the MEDACIS + DHRD group stratified based on consent, nonconsent, and noneligibility. Here, intergroup comparisons were performed on age, gender, ACS diagnosis, and the primary outcome of psychological outcomes (Table 2). As a supplementary analysis, intergroup comparisons were performed on an extensive list of patient demographics (supplementary S2). At the third and final level, we performed intergroup comparison by comparing age, gender, ACS diagnosis, and psychological outcomes between patients excluded based on different eligibility criteria. No statistical adjustment for confounding was performed within the statistical analysis, and all comparisons are crude comparisons. However, to explore the effect of each individual eligibility criterion, several sensitivity analyses of comparison between noneligible patients and the patients excluded based on either current/previous antidepressant treatment, coronary artery bypass grafting, EGFR, or transaminases, or participation in another trial. No statistical approach to handle missing data was applied in the current study. No missing data were present regarding age and gender, and the level of and reasons for missing data on the HADS is within the manuscript. All statistical analysis was performed using SAS Enterprise version 9.4 (Proprietary Software 9.4, SAS Institute, Inc., Cary, NC USA), and a 5% level of significance was chosen.

**TABLE 1:** Baseline characteristics of population in the study

Baseline characteristics based on origin			
	DHRD (*n* = 282)	MEDACIS + DHRD (*n* = 758)	MEDACIS (*n* = 462)
Age, years mean (*SD*)	63.7 (10.6)	64.3 (11.8)	72.5 (13.7)
Male	74.1	73.6	65.6
STEMI *n* (%)	83 (29.4)	270 (35.6)	115 (25.9)
Non‐STEMI	113 (40.0)	435 (57.4)	320 (69.3)
UAP	86 (30.5)	53 (7.0)	27 (5.8)

Intergroup comparison		
	DHRD versus. MEDACIS + DHRD	MEDACIS + DHRD versus. MEDACIS
Age, mean diff. years (95% CI)	−0.57 (−2.15; 1.00) *p* > .10	−8.12 (−9.6;−6.66) *p* < .0001
Male %	74.1 versus. 73.6 *p* > .10	73.6 versus. 65.6 *p* = .003
ACS diagnosis	*p* < .0001	*p* = .0002

Note

Table of baseline demographics and intergroup comparisons for cohorts based on origin.

Abbreviations: ACS; acute coronary syndrome, CI; confidence interval; DHRD, Danish heart rehabilitation database, *SD*; standard deviation, STEMI: ST elevation myocardial infarction, UAP; unstable angina pectoris.

**TABLE 2:** Characteristics of cohort included in both the MEDACIS and the DHRD databases

Demographic	Participants (*n* = 252)	Non‐consent patients (*n* = 144)	Participants versus. Non‐consent	Non‐eligible patients (*n* = 362)	Participants versus. non‐eligible
Age, mean (*SD*)	62.4 (11.1)	65.5 (12.0)	−3.07 (−5.42;−0.72), *p* = .02	65.2 (12.1)	−2.81 (−4.70; −0.92), *p* = .004
Male	196 (77.7)	96 (66.7)	*p* = .02	266 (73.5)	*p* > .10
STEMI, *n* (%)	109 (43.25)	54 (37.5)	*p* > .10	107 (29.6)	*p* = .0005
Non‐STEMI, *n* (%)	131 (52.0)	86 (59.7)		218 (60.2)	
UAP, *n* (%)	12 (4.76)	4 (2.8)		37 (10.2)	
Psychological outcomes					
HADS‐A score, mean (*SD*)	2.73 (2.8)	3.64 (4.0)	−0.90 (−1.66;−0.16) *p* = .02	4.39 (3.9)	−1.81 (−2.35;−1.27), *p* < .0001
HADS‐A ≥ 8, *n* (%)	15 (6.0)	19 (20.0)	*p* = .0004	56 (21.9)	*p* < .0001
HADS‐D score, mean (*SD*/95% CI)	1.85 (2.32)	2.65 (3.1)	−0.80 (−1.41;−0.20), *p* = .009	3.66 (3.7)	−1.65 (−2.25;−1.06), *p* < .0001
HADS‐D ≥ 8, *n* (%)	14 (5.6)	8 (8.4)	*p* > .10	40 (15.6)	*p* = .0003

Note

Table of baseline demographics and intergroup comparisons for MEDACIS and DRHD patients divided based on eligibility and consent.

Abbreviations: ACS; acute coronary syndrome, CI; confidence interval, DHRD; Danish heart rehabilitation database, HADS‐A/D; hospital anxiety and depression scale—anxiety/depression, *SD*; standard deviation, STEMI; ST elevation myocardial infarction, UAP; unstable angina pectoris.

## 4 RESULTS

### 4.1 MEDACIS versus DHRD

Participants had an average age of 63.7, 64.3, and 72.5 years in the DHRD (*n* = 282), MEDACIS + DHRD (*n* = 758), and MEDACIS groups (*n* = 462), respectively (Table 1). Similarly, 74.1%, 73.6%, and 65.6% of the population were males, respectively. Intergroup comparisons showed no difference in age and gender between the DRHD and MEDACIS + DHRD groups. However, the MEDACIS group was significantly older (8.1 years) and more likely to be females compared to the MEDACIS + DHRD group. The ACS case mix in MEDACIS + DHRD was significantly different compared to the remaining two groups.

### 4.2 MEDACIS + DHRD

#### 4.2.1 Participants versus noneligible versus. nonconsent

Compared to the MEDACIS participants, noneligible patients were different regarding the predominance of demographic variables (supplementary S2). Noneligible patients were, on average, 2.8 years older (95% CI 0.9; 4.7) and were more likely to suffer from unstable angina pectoris (UAP) (Table 2). They were less likely to be married and had a shorter education. They also showed more comorbidity with a higher frequency of heart valve disease, diabetes, kidney disease, mental disorder, and malignancy. Based on current medication, they were more likely to be on antidepressants, insulin, and diuretics but less likely to be in treatment with angiotensin‐II antagonists (supplementary S2).

Compared to MEDACIS participants, nonconsenting eligible patients were, on average, 3.1 years older (95% CI 0.7; 5.4) and more likely to be females (Table 2). Furthermore, nonconsenting patients suffered more from atrial fibrillation, more frequently had a pacemaker, and were more likely to be on ACE inhibitors (supplementary S2). Otherwise, nonconsenting patients were comparable to participants in the MEDACIS trial.

### 4.3 Psychological outcomes

Comparing participants versus. noneligible patients showed pronounced differences in depressive symptoms and anxiety between groups. As continuous score, noneligible patients, on average, had 1.81 points (95% CI 1.27; 2.35, *p* < .0001) higher HADS‐A and 1.65 points (95% CI 1.06; 2.25, *p* < .0001) higher HADS‐D. Applying relevant cutoff values, noneligible patients had significantly more depressive symptoms and anxiety (15.6 versus. 5.6%, *p* = .0003 and 21.6 versus. 6.0%, *p* < .0001, respectively).

Participants and nonconsenting patients differed on depressive symptoms and anxiety. Looking at the HADS as a continuous score, nonconsenting patients, on average, had 0.90 points (95% CI 0.16; 1.66, *p* = .02), higher HADS‐A and 0.80 points (95% CI 0.2; 1.41, *p* = .01), and higher HADS‐D (Table 2). Applying relevant cutoff values, nonconsenters had significantly more anxiety (20.0 versus. 6.0%, *p* = .0004).

In the MEDACIS participant data, 0.4% of HADS data were missing; in the nonconsent group, 34% were missing HADS and in the noneligible groups 29.3% (Table 3).

**TABLE 3:** Reasons for missing HADS data in the cohort identified in both the MEDACIS and the DHRD databases

Reason for missing	Participants (*n* = 252)	Nonconsent (*n* = 144)	Noneligible (*n* = 362)	Total (*n* = 758)
HADS not delivered				
Not fluent in Danish	0	1	4	5
Patient did not want to fill out	0	6	5	11
Known or current psychiatric diagnosis	0	0	18	18
Unknown reason	0	17	33	51
HADS delivered, no score available				
Patient did not return HADS	1	23	38	61
Returned but score not reported	0	1	4	5
Patient discontinued rehabilitation	0	1	4	5
Total *n*	1	49	106	156
% missing data	0.4	34.0	29.3	**20.6**

Note

Reasons for missing data of the HADS data.

### 4.4 Sensitivity analyses

In an effort to explore the effect of specific eligibility, sensitivity analyses were performed comparing noneligible patients excluded due to a specific criterion to the remaining noneligible patients on age, gender, and psychological outcomes (Table 4 a‐d).

**TABLE 4:** a‐d Sensitivity analysis on noneligible participants

4a: Participants excluded for current or previous antidepressant treatment (*n* = 82)			
Variable	Noneligible	Excluded	Group difference
Age, years (*SD*/95% CI)	66.5 (11.7)	60.7 (12.6)	5.8 (2.8;8.7) *p* = .0003
Gender, male (%)	77.9	58.5	*p* = .001
HADS‐A score, mean (*SD*)	3.82 (3.61)	6.45 (4.39)	−2.60 (−3.80;−1.50) *p* < .0001
HADS‐A ≥ 8, %	17.0	39.29	*p* = .0008
HADS‐D score, mean (*SD*)	3.18 (3.07)	5.39 (4.99)	−2.20 (−3.30;−1.20) *p* < .0001
HADS‐D ≥ 8, %	11.0	32.14	*p* = .0003
4b: Participants excluded for coronary artery bypass graft (*n* = 75)			
Age, years (*SD*/95% CI)	64.7 (12.7)	67.3 (9.4)	−2.6 (−5.7;0.5) *p* = .099
Gender, male (%)	71.1	82.7	*p* = .05
HADS‐A score, mean (*SD*)	4.6 (4.1)	2.7 (2.7)	2.2 (1.00;3.30) *p* = .0003
HADS‐A ≥ 8, %	26.2	5.5	*p* = .0007
HADS‐D score, mean (*SD*)	3.97 (3.89)	2.52 (2.49)	1.45 (0.30;2.50) *p* = .01
HADS‐D ≥ 8, %	18.8	3.7	*p* = .005
4c: Participants excluded for EGFR or Transaminases (*n* = 25)			
Age, years (*SD*/95% CI)	64.7 (12.0)	72.7 (11.6)	−8.1 (−12.9;3.2) *p* > .10
Gender, male (%)	73.6	72.0	*p* > .10
HADS‐A score, mean (*SD*)	4.40 (3.95)	4.30 (3.92)	0.09 (−1.63;1.80) *p* > .10
HADS‐A ≥ 8, %	21.9	21.7	*p* > .10
HADS‐D score, mean (*SD*)	3.67 (3.72)	3.61 (3.31)	−0.06 (−1.50;1.60) *p* > .10
HADS‐D ≥ 8, %	14.6	26.1	*p* > .10
4d: Participants excluded for participation in another trial (*n* = 37)			
Age, Years (*SD*/95% CI)	65.3 (12.4)	64.4 (10.0)	0.9 (−3.2;5.1) *p* > .10
Gender, Male (%)	72.0	86.5	*p* = .08
HADS‐A score, mean (*SD*)	4.42 (3.95)	4.15 (3.86)	0.27 (−1.30;1.90) *p* > .10
HADS‐A ≥ 8, %	22.7	14.8	*p* > .10
HADS‐D score, mean (*SD*)	3.73 (3.75)	3.04 (3.04)	−0.70 (−0.80;2.20) *p* > .10
HADS‐D ≥ 8, %	17.0	3.7	*p* = .09

Note

Sensitivity analysis based on eligibility criteria.

Abbreviation: HADS‐A/D; hospital anxiety and depression scale—anxiety/depression.

Patients excluded due to current or previous antidepressant treatment were more likely to be younger and females (Table 4a). They had 2.6 and 2.8 points higher HADS‐A/D scores compared to other noneligible patients, respectively.

Patients excluded due to coronary artery bypass grafting were not different with regard to age and gender (Table 4b). They had 2.2 and 1.45 points lower HADS‐A/D scores compared to other noneligible patients, respectively.

Patients excluded due to reduced EGFR or increased transaminases and participation in another trial were not different with regard to age and gender (Table 4c,d). Furthermore, they did not differ on psychiatric outcomes compared to other noneligible patients.

## 5 DISCUSSION

### 5.1 Key results

Noneligible patients had significantly higher levels of depressive symptoms and anxiety and were older and more likely to suffer from UAP. Furthermore, they were less likely to be married, had a lower educational level, were more likely to have multimorbidity, and used more medication. Nonconsenting patients had significantly higher levels of depressive symptoms and anxiety and were older more likely to be females compared to participants. Sensitivity analysis of individual eligibility criteria showed that patients excluded based on current or previous antidepressant treatment had higher levels of both anxiety and depression, whereas patients excluded based on CABG treatment had lower levels of anxiety and depression.

### 5.2 Interpretation

The current study gives a unique insight into different aspects of the external validity of a recently performed randomized clinical trial in the field of depression following ACS. Especially problematic were the significant differences between participants and eligible nonconsenting patients, who were older and more likely to be females. Eligible nonparticipants in a similar trial have also previously been shown to be older; however, the overrepresentation of females was not shown (Hansen et al., 2011). Based on a combined analysis of several large Canadian prospective multicenter cohort studies of patients with ACS, it was demonstrated that participants in clinical trials were younger, more frequently men, and had fewer comorbidities (Hutchinson‐Jaffe et al., 2010). Increasing age and female gender have also been shown to be associated with nonparticipation in observational studies in ACS (Grace et al., 2004; Sorensen et al., 2005). Differences in age and gender among participants and nonparticipants are highly relevant in Danish patients with a recent ACS since they have been shown to be associated with increased risk of developing depression (Joergensen et al., 2016; Osler et al., 2016). Furthermore, these eligible nonconsenting patients had significantly higher symptoms of depression and anxiety. It is problematic that seemingly eligible patients opted not to participate in the MEDACIS trial, especially as the primary outcome was to prevent the development of depressive symptoms. Essentially, this is an evidence that the MEDACIS trial suffered from healthy‐participant bias.

Another important aspect of the results was the significantly higher levels of depressive symptoms and anxiety, more somatic comorbidity, and higher age in the noneligible patients. This shows, not surprisingly, that eligibility determined by researchers before initiation of a given trial heavily affects the external validity of the trial's results. Given the large difference between the participants and noneligible patients, the results of the MEDACIS trial should be deemed of very low external validity to noneligible patients. Furthermore, in light of the few events within the MEDACIS trial, it cannot be excluded that the intervention tested within the MEDACIS trial would have had an effect in the noneligible patients as well as in the nonconsent patients.

Lastly and interestingly, the sensitivity analysis of the exclusion criteria based on current and/or previous antidepressant treatment significantly altered the levels of depressive symptoms and anxiety within the noneligible participants. Hence, this one exclusion criterion seems to interact with the measurement of the primary outcome of the trial. Unfortunately, we cannot discriminate based on whether it was current or previous antidepressant treatment driving this difference. This would be particularly interesting seeing that the purpose of the trial was the prevention of depressive symptoms.

### 5.3 Limitations

The first limitation of the study is the discrepancy between the MEDACIS screening list and the cohort from the DHRD. A likely explanation for individuals found only in the DHRD database is that the MEDACIS trial did not screen during the weekends; hence, patients could be discharged before contact with the study investigators. Similarly, individuals unique to the MEDACIS screenings list are due to CR not being mandatory (i.e., patient option out); hence, the presence in DHRD comprises only patients who choose to participate in CR.

Secondly, the timing of the collection of the HADS was performed according to the practice of the individual outpatient clinic. This results in some variability in the timing since some outpatient clinics collect the HADS at the initial meeting and others at the end of CR. A further important limitation of the HADS data is the presence of missing data from the DHRD. In the DHRD, information on screening for depression performed (yes/no) during the conduct of the MEDACIS trial was a key indicator; however, the HADS scoring was not mandatory, and therefore, there are considerable amounts of missing data.

### 5.4 Strengths

The strength of this is concurrent data collection within the same geographical region with data at the patient level. In essence, the design of the current study is equivalent to a prospective cohort study with a nested randomized controlled trial, which previously was conducted within the field of psychiatric research following acute coronary syndrome (Kang et al., 2015; Kim et al., 2014).

The current study could be seen as an exploration of the selection process that is related to the conduct of clinical trials. A distinction between biases related to the researcher's choices (noneligible patient) and patient's choice (nonconsenting patients) was made in order to distinguish levels of this selection process further. In making this discrimination, we hoped to describe different dimensions of the external validity of a randomized clinical trial and be able to test it quantitatively.

### 5.5 Generalizability

Much literature highlights the limitations of external validity in both randomized clinical trials and observational studies in general (Dekkers et al., 2010; Rothwell, 2005; Smyth et al., 2019) and in the field of acute coronary syndrome research (Grace et al., 2004; Hansen et al., 2011; Hutchinson‐Jaffe et al., 2010; Smyth et al., 2019; Sorensen et al., 2005). The current study adds to this literature, where it repeatedly has been shown that such trials are more likely to include healthier younger males. Specifically, for the current trial, the discrimination between participants, eligible nonparticipants, and noneligible patients sheds novel light on distinctions between these distinct groups. The results regarding differences in baseline levels of symptoms of depression and anxiety are highly relevant in the field of psychosomatic research in cardiology, which holds several Cochrane reviews (Baumeister et al., 2011; Richards et al., 2017). When participation in a trial is associated with the primary outcome, special care needs to be incorporated when designing clinical trials. A possible applied design to ascertain data on this issue would be to conduct a prospective cohort study with continuous follow‐up and within the same cohort perform a nested randomized clinical trial.

As could be expected, differences were present between noneligible patients and participants; however, more troublingly significant differences were shown between nonconsenting patients and participants. The presence of depressive symptoms and anxiety has a significant impact on patients’ willingness to give informed consent in clinical trials and, therefore, represents a serious threat to external validity. Future clinical trials investigating prevention of psychological outcomes should apply as few exclusion criteria as possible to increase external validity.

### PEER REVIEW

The peer review history for this article is available at https://publons.com/publon/10.1002/brb3.2132.

## Supporting information

Supplementary Material

## Data Availability

The data are not publicly available due to their containing information that could compromise the privacy of research participants.
