# Supplementary material for: External validity of randomized clinical trial studying preventing depressive symptoms following acute coronary syndrome
Source: Brain Behav. 2021 Jun 17;11(8):e02132. doi: 10.1002/brb3.2132 (PMC8413812; doi:10.1002/brb3.2132)
Supplement: Supplementary file 1 — Supplementary Material [file BRB3-11-e02132-s001.docx]

**Supplementary material**

**F1** Recruiting centres


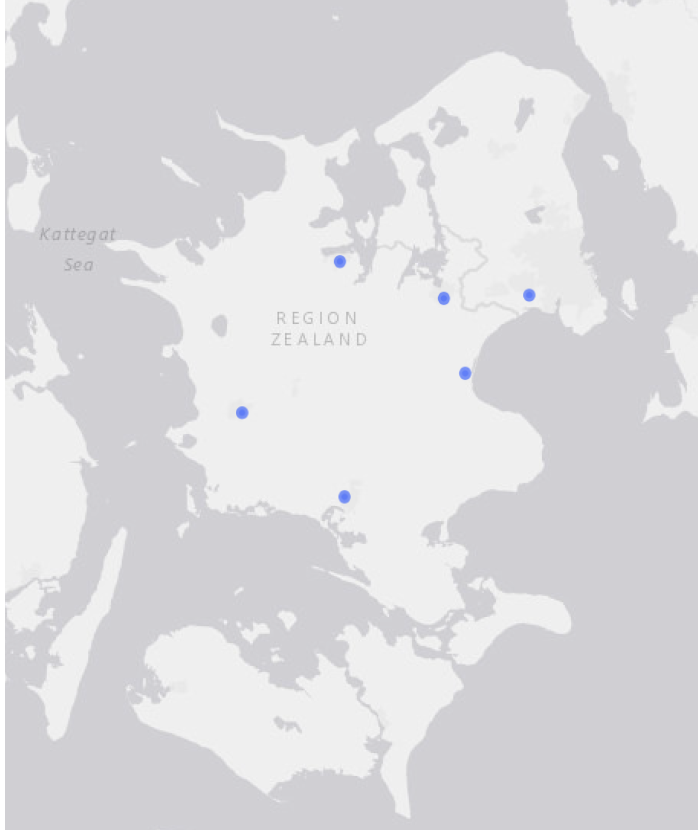


Legend: Including centres of the MEDACIS trial. Departments of cardiology at Køge, Roskilde, Holbaek, Slagelse/Næstved and Hvidovre hospitals.

**S1 Recruitment divided by centre**

| Centre | Screening start | Screening end | Months | Recruited pr. centre |
| --- | --- | --- | --- | --- |
| Koege hospital | 18 JAN 2016 | 10 MAY 2017 | 16 | 72 |
| Roskilde hospital | 09 FEB 2016 | 24 APR 2017 | 14.5 | 30 |
| Holbaek hospital | 01 MAR 2016 | 18 APR 2017 | 13.5 | 60 |
| Hvidovre hospital | 22 AUG 2016 | 06 MAR 2017 | 6.5 | 12 |
| Slagelse hospital  Naestved hospital | 03 AUG 2016 | 20 APR 2017 | 8.5 | 78 |
| Total | - | - | 59 | 252 |

Legend: Screening timing, period, and number of participants divided by including centre.

**S2** Characteristics of participants within both the MEDACIS and DHRD databases

| **Demographic** | Participants (n=252) | Non-consent patients  (n=144) | Participants vs.  Non-consent | Non-eligible patients (n=362) | Participants vs.  non-eligible |
| --- | --- | --- | --- | --- | --- |
| **Civil status (%)** | | | | | |
| Unmarried | 24 (10.1) | 19 (13.8) | P>0.10 | 44 (12.9) | P=0.01 |
| Married | 173 (73.0) | 89 (65.0) |  | 203 (59.5) |  |
| Divoreced | 24 (10.1) | 15 (11.0) |  | 54 (15.8) |  |
| Widowed | 15 (6.3) | 12 (8.8) |  | 36 (10.6) |  |
| **Educational level (%)** | | | | | |
| Primary school | 32 (13.5) | 9 (7.38) | P>0.10 | 49 (15.8) | P=0.0001 |
| Short courses | 8 (3.4) | 10 (8.2) |  | 39 (12.6) |  |
| High school education | 107 (45.1) | 59 (48.4) |  | 127 (41.0) |  |
| Some college | 23 (9.7) | 10 (8.2) |  | 19 (6.1) |  |
| Bachelor’s degree | 41 (17.3) | 22 (18.0) |  | 40 (12.9) |  |
| Master’s degree | 23 (9.7) | 10 (8.2) |  | 20 (6.5) |  |
| Other | 2 (0.8) | 2 (1.6) |  | 16 (5.1) |  |
| **Smoking (%)** | | | | | |
| Never smoked | 71 (30.0) | 48 (36.4) | P>0.10 | 107 (31.2) | P>0.10 |
| Recently stopped | 96 (40.5) | 45 (34.1) |  | 126 (36.7) |  |
| Former smoker | 27 (11.4) | 17 (12.9) |  | 42 (12.2) |  |
| Current smoker | 42 (17.7) | 22 (16.67) |  | 67 (19.5) |  |
| Systolic blood pressure, mmHg (SD) | 135.5 (15.5) | 138.2 (17.3) | 0.4 (-1.9 ; 2.7) P>0.10 | 134.4 (17.4) | 1.2 (-1.7;4.1), P>0.10 |
| Diastolic blood pressure, mmHg (SD) | 80.03 (9.8) | 79.6 (11.5) | -2.8 (-6.3;0.7) P>0.10 | 78.3 (9.9) | 1.8 (0.1;3.5), P=0.041 |
| **Comorbidity** (%) | | | | | |
| Heart valve disease | 1 (0.62) | 3 (2.1) | P>0.10 | 15 (4.3) | P=0.03 |
| Heart failure | 26 (11.0) | 14 (9.9) | P>0.10 | 46 (13.2) | P>0.10 |
| Diabetes | 25 (10.6) | 21 (15.0) | P>0.10 | 85 (24.4) | P<0.0001 |
| Atrial fibrillation | 3 (1.9) | 10 (7.0) | P=0.02 | 18 (5.2) | P=0.09 |
| Pacemaker | 0 (0) | 4 (2.8) | P=0.05 | 2 (0.6) | P>0.10 |
| ICD | 1 (0.62) | 1 (0.7) | P>0.10 | 5 (1.4) | P>0.10 |
| Stroke/TCI | 1 (0.62) | 5 (3.5) | P=0.10 | 11 (3.2) | P>0.10 |
| Peripheral atherosclerosis | 0 (0) | 1 (0.7) | P=0.05 | 8 (2.3) | P=0.06 |
| Kidney disease | 0(0) | 0 (0) | P>0.10 | 12 (3.4) | P<0.01 |
| Musculoskeletal disease | 6 (3.7) | 14 (10.0) | P=0.04 | 45 (12.9) | P=0.0008 |
| Mental disorder | 1 (0.62) | 3 (2.1) | P>0.10 | 29 (8.3) | P<0.0001 |
| Malignant disease | 2 (1.3) | 1 (0.7) | P>0.10 | 7 (2.0) | P<0.0001 |
| COPD | 4 (2.5) | 1 (0.7) | P>0.10 | 20 (5.7) | P>0.10 |
| **MEDICATION, n (%)** | | | | | |
| Current antidepressant | 0 (0) | 0 (0) | P>0.10 | 60 (17.2) | P=<0.0001 |
| Aspirin | 180 (76.0) | 112 (78.9) | P>0.10 | 257 (73.6) | P>0.10 |
| Thrombocyte inhibitor | 177 (74.7) | 103 (72.5) | P>0.10 | 235 (67.3) | P=0.07 |
| Other anticoagulant | 12 (5.1) | 14 (10.0) | P=0.09 | 24 (6.9) | P>0.10 |
| Statins | 180 (75.9) | 108 (76.1) | P>0.10 | 275 (78.8) | P>0.10 |
| No-statin Cholesterol lowering drugs | 11 (4.6) | 8 (5.6) | P>0.10 | 7 (2.0) | P=0.09 |
| Beta-blockers | 146 (61.6) | 84 (59.2) | P>0.10 | 1 (0.3) | P>0.10 |
| Oral antidiabetics | 18 (7.6) | 5 (3.5) | P>0.10 | 35 (10.0) | P>0.10 |
| Insulin | 5 (2.1) | 2 (1.4) | P>0.10 | 25 (7.2) | P=0.007 |
| Calcium antagonists | 55 (23.2) | 36 (25.4) | P>0.10 | 97 (27.8) | P>0.10 |
| Long acting nitrates | 14 (5.9) | 5 (3.5) | P>0.10 | 20 (5.7) | P>0.10 |
| Nitro-glycerine – discretionary | 109 (46.0) | 49 (34.5) | P=0.03 | 137 (39.3) | P>0.10 |
| Other angina medication | 4 (1.7) | 1(0.7) | P>0.10 | 4 (1.2) | P>0.10 |
| ACE inhibitors | 91 (38.4) | 71 (50.0) | P=0.03 | 157 (45.0) | P>0.10 |
| Angiotensin-II antagonists | 14 (5.9) | 2 (1.4) | P=0.004 | 4 (1.4) | P=0.004 |
| Diuretics | 64 (27.0) | 43 (30.3) | P>0.10 | 126 (36.1) | P=0.025 |
| Anti-arrhythmic treatment | 1 (0.4) | 1 (0.7) | P>0.10 | 5 (1.4) | P>0.10 |
